# The Promise and Challenges of CAD‐Assisted Diagnosis in Achalasia

**DOI:** 10.1111/den.15076

**Published:** 2025-06-25

**Authors:** Niroshan Muwanwella, Krish Ragunath

**Affiliations:** ^1^ Curtin Medical School Curtin University Perth Australia; ^2^ Department of Gastroenterology Royal Perth Hospital Perth Australia

Since its inception 60 years ago [1], Artificial Intelligence (AI) has undergone complex evolvement. The last decade has seen its most rapid advancement, and AI is now an essential part of everyday life. The introduction of computer‐aided diagnosis (CAD) to the realm of endoscopy brings a paradigm shift to medical diagnostics. Two of the most important applications of AI in endoscopy are computer‐aided detection (CADe) and computer‐aided diagnosis (CADx) [2]. Both these applications are developed by training AI models to detect vascular and mucosal patterns on mucosal lesions. However, in conditions such as achalasia, where specific lesions are not seen, this model of detection will not work. Therein lies a good reason for the study developed by Shiwaku and colleagues, which marks a different phase of CAD [3].

The study under discussion presents compelling evidence of CAD's potential to enhance the sensitivity and specificity of achalasia diagnosis, particularly among non‐expert endoscopists. These findings highlight a transformative step in the democratization of medical expertise, where technology bridges the gap between novice and experienced clinicians. However, as with any innovation, the translation of these results into real‐world clinical practice warrants careful scrutiny.

One of the most striking outcomes of this study is the dramatic improvement in sensitivity among inexperienced endoscopists, rising from 66.9% to 91.9% with CAD assistance. Even experienced practitioners benefited from CAD, with their sensitivity increasing from 79.5% to 90.8%. These findings suggest that CAD has the potential to mitigate diagnostic variability and significantly reduce the rate of missed achalasia cases. Given the availability of highly effective treatments, such as peroral endoscopic myotomy (POEM), improving early and accurate diagnosis is undoubtedly beneficial for optimizing patient outcomes.

Yet, despite these promising results, significant questions remain regarding CAD's application in real‐world clinical settings. The study's limitations underscore certain critical challenges. Achalasia is a rare disease, and the artificially high prevalence of cases in this study (50% of the sample) does not reflect the reality of routine endoscopic practice. In an actual clinical setting, where achalasia cases are far less frequent, the performance of CAD may differ. The concern here is whether CAD will maintain its diagnostic power when faced with the complexities and unpredictability of daily endoscopic practice, where the pre‐test probability of achalasia is much lower.

Another point of concern is the reliance on pre‐selected video data, captured by a single expert. While this controlled setting ensures consistency in the dataset, it does not account for the real‐life variability in endoscopic image quality, patient presentation, and operator technique. Additionally, the study does not address potential biases introduced by manometry‐confirmed cases, as not all non‐achalasia cases underwent confirmatory testing. This raises questions about the true specificity of CAD in broader practice.

Moreover, the psychological impact of CAD on clinical decision‐making warrants further exploration. The study highlights that endoscopists were inclined to alter their initial diagnosis based on CAD suggestions, often converting false‐negative diagnoses into true positives. While this suggests a productive human‐machine synergy, it also raises concerns about over‐reliance on AI‐based recommendations. Will endoscopists, particularly those with less experience, begin to defer excessively to CAD, potentially leading to diagnostic complacency or even automation bias? This phenomenon, called automation bias (AB) was initially investigated in the aviation sector [4]. With the advent of AI integration into health care, this is increasingly being studied, with researchers and clinicians paying increasing attention to it in the last decade or so. In an earlier study, it was found that in 6% of cases, clinicians overrode correct decisions in favor of erroneous ones from AI [5]. A more recent review found that AB, also referred to as automation‐induced complacency and confirmation bias, affects both naïve and expert participants and that it cannot be prevented by training or instructions [6]. This, along with alarm fatigue, needs to be considered carefully when incorporating CAD systems into everyday endoscopic practice [7].

In addition to this, the position statement on AI in colonoscopy of the World Endoscopy Organization acknowledged that whilst CADe systems for colonoscopy were likely to improve efficiency by reducing adenoma miss rates in the short term, that they were likely to increase health care costs by detecting more adenomas. High quality research was advocated to investigate whether these notions hold true in the wider clinical practice in different health care systems [8]. This statement holds true for all the AI systems in endoscopy beyond CADe and CADx systems colonoscopy including the system used in the study under discussion. In their discussion on quality indicators in endoscopic screening and the role of AI, Okamoto and Hirasawa argue that AI faces the challenge of lower accuracy in atypical presentations or low quality images [9]. This is the exact issue facing incorporating AI in rare disorders with no specific endoscopic lesions such as achalasia.

Beyond the scope of achalasia, this study is a valuable proof of concept for the use of AI in diagnosing rare diseases. The ability of CAD to permanently store and share diagnostic insights across institutions holds great promise for medical knowledge dissemination. However, achieving seamless integration into clinical workflows will require addressing several logistical and ethical considerations, including regulatory approval, cost‐effectiveness, and ensuring that AI tools enhance, rather than replace, human expertise.

In conclusion, this study represents an exciting step forward in AI‐assisted diagnostics, demonstrating that CAD can significantly improve the detection of achalasia. However, before widespread clinical adoption, further validation in real‐world settings is essential. The medical community must ensure that CAD not only augments diagnostic accuracy but also integrates effectively within the nuanced complexities of clinical practice. Ultimately, technology should serve as a tool to empower clinicians, rather than dictate and unduly influence their judgment.

By far, the greatest danger of Artificial Intelligence is that people conclude too early that they understand it. (Eliezer Yudkowsky)

## Author Contributions

**Niroshan Muwanwella:** conceptualization (equal); writing – original draft (lead). **Krish Ragunath:** conceptualization (equal); writing – review and editing (lead).

## Conflicts of Interest

Prof Krish Ragunath: Associate Editor of Digestive Endoscopy. The other author declares no conflicts of interest.

## Linked Articles

Computer‐aided detection for esophageal achalasia (with video) https://doi.org/10.1111/den.15028.
